# Reckless Midwifery

**Published:** 1882-12

**Authors:** 


### RECKLESS MIDWIFERY.

A horrible death has occurred at the village of Eye, near Petersborough. A woman was attended in her labor by two neighbors, one of whom professed to be a midwife, the other a nurse. She was delivered shortly after midnight, on Saturday last, of a living child; and when Mr. Beecroft, a retired surgeon living close by, was called in soon after delivery, he found the unfortunate woman dead, with her uterus, one ovary, and fallopian tube lying in the bed beside her, these parts having been actually torn or cut away by the women in attendance.—*British Medical Journal.*

